# Blinding Bilateral Hyperviscosity Retinopathy in a 43-Year-Old Nigerian Male with Lymphoplasmacytic Lymphoma: A Case Report and Management Challenges

**DOI:** 10.1155/2014/567632

**Published:** 2014-05-05

**Authors:** Abdulkabir A. Ayanniyi, Uchenna Godswill Ejikeme, Yohanna Tanko, Rilwan C. Muhammad, Obiageli E. Nnodu

**Affiliations:** ^1^Department of Ophthalmology, College of Health Sciences, University of Abuja, PMB 117, Abuja, Nigeria; ^2^Department of Ophthalmology, University of Abuja Teaching Hospital, Gwagwalada, Abuja, Nigeria; ^3^Department of Haematology and Blood Transfusion, University of Abuja Teaching Hospital, Gwagwalada, Abuja, Nigeria; ^4^Department of Haematology and Blood Transfusion, College of Health Sciences, University of Abuja, Gwagwalada 90003, Nigeria

## Abstract

Lymphoplasmacytic lymphomas are rare and may present with uncommon and devastating symptoms. We report a case of a 43-year-old male who presented with bleeding gums and sudden onset of bilateral blindness but was not on anticoagulants and had no family history of bleeding disorder. He had bilateral hyperpigmented infraorbital skin lesions, visual acuities (VA) of hand motion in both eyes (blindness), round and sluggish pupils, and bilateral diffuse and extensive retinal haemorrhages obliterating the retinal details with central visual field defects. The optical coherence tomography revealed retinal haemorrhage, oedema, detachment, and diffuse photoreceptors damage. Investigations revealed elevated ESR and *β*_2_ microglobulin, monoclonal peak on serum protein electrophoresis, high IG with lambda restriction on serum, and urine immunofixation with increased lymphocytes and plasma cells in the bone marrow. A diagnosis of lymphoplasmacytic lymphoma complicated by blinding hyperviscosity retinopathy was made. In the absence of an aphaeresis machine, he received four cycles of manual exchange blood transfusion (EBT) and commenced with chlorambucil/prednisolone due to difficulty in obtaining blood for continued EBT. His general condition and VA has improved and he is stable for more than six months into treatment.

## 1. Introduction

Lymphoplasmacytic lymphoma is a B-cell neoplasm affecting the bone marrow, spleen, or lymphoid tissues and usually is associated with IgM type paraprotein production resulting in hyperviscosity but our patient had peak IgG paraprotein [1–3]. Hyperviscosity retinopathy is visually impairing, mimics central retinal vein occlusion, is related to dysproteinaemia such as Waldenstrom macroglobulinemia or multiple myeloma, and is often reversible by plasmapheresis [4–8]. As our hospital does not have an aphaeresis machine, our patient received four cycles of manual EBT followed by chlorambucil due to cost considerations. Lymphoplasmacytic lymphoma with hyperviscosity from high IgG paraprotein resulting in bilateral hyperviscosity retinopathy is uncommon. Our patient illustrates the challenge of providing basic diagnostic and support services for cancer patients in resource poor settings which requires concerted effort to overcome.

## 2. Case Presentation

**Figure 1:**
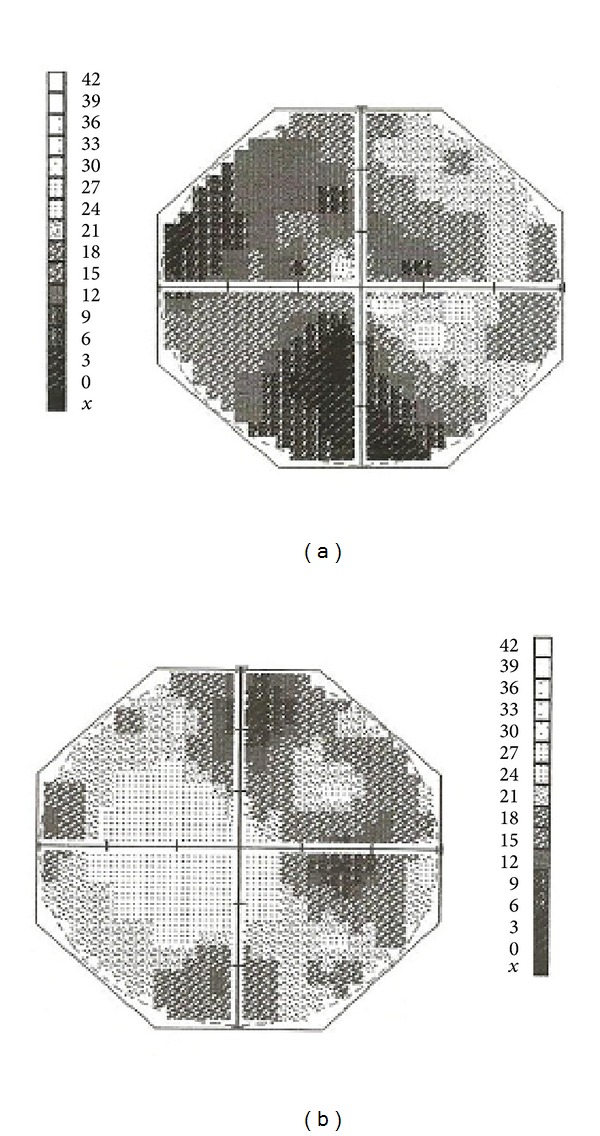
Central visual field defects: (a) the right eye; (b) the left eye.

A 43-year-old Nigerian male presented with history of pain and bleeding from the gums for three months and bilateral visual loss of six weeks duration. The bleeding was heavy and in clots with associated generalized body weakness and increased exercise intolerance. There was neither history of trauma, tooth extraction, nor treatment with medications known to encourage bleeding such as warfarin or heparin. The patient had neither family history of bleeding disorder nor past history of haemoptysis, epistaxis, haematuria, melaena stool, and easy bruising. The visual loss was sudden, progressive, and essentially painless, with the left eye (LE) being first affected followed by the right eye (RE) about a month after. The patient denied any history of eye disease or diabetes mellitus in self or family. He neither smoked cigarettes nor imbibed alcohol. He was found to have mild hypertension elsewhere shortly before presentation but had not been placed on any antihypertensive drug. The general examination revealed a middle-aged man, mildly pale, and essentially in stable general clinical state. The respiratory rate was 22 cycles/min, pulse rate was 136 beats/min, and BP was 140/90 mmHg. The essential ocular findings were bilateral hyperpigmented infraorbital skin lesions, visual acuities (VA) of hand motion in both eyes, round and sluggish pupils, and diffuse and extensive retinal haemorrhages obliterating the retinal details. The intraocular pressure was 12 mmHg in both eyes. There were central visual field defects, worse in the LE (Figure 1).

**Figure 2:**
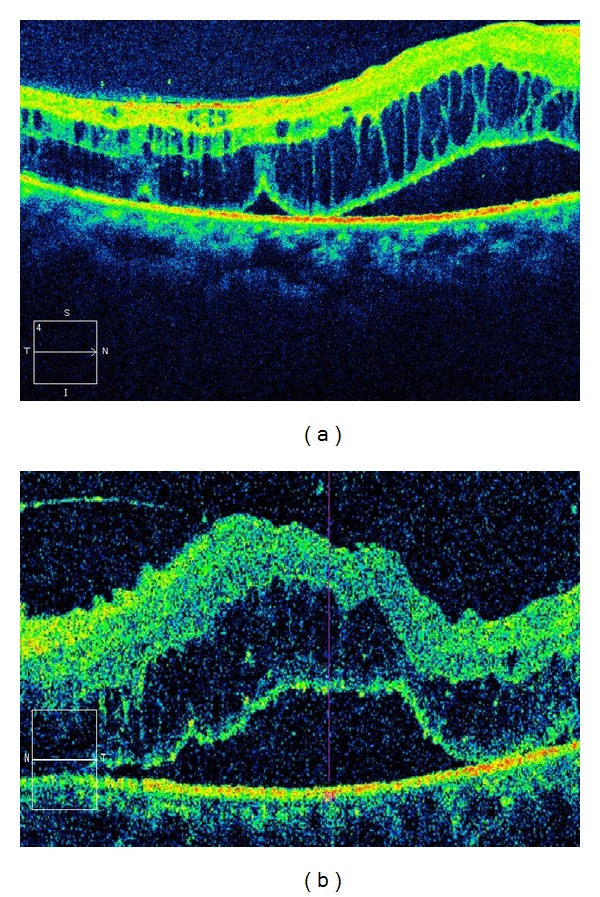
Optical coherence tomography: (a) the right eye; (b) the left eye.

The optical coherence tomography revealed retinal haemorrhage, oedema, detachment, and diffuse photoreceptors damage (Figure 2).

The results of initial blood investigations showed reduced Na^+^, bicarbonate, and alkaline phosphatase and elevated creatinine (Table 1).

The coagulation studies showed elevated PT and PTTK results with INR = 1.42 (0.9–1.3). The serum protein electrophoresis revealed a large monoclonal peak (75.1 g/dL) in the *β*-region which was shown to be IgG Lambda by serum and urine immunofixations. *β*_2_-microglobulin was elevated −7.1 (1.16–2.52 mg/dL) with normal lactate dehydrogenase. The bone marrow examination showed a cellular marrow with reduced megakaryopoiesis, myelopoiesis, and erythropoiesis and increased lymphocytes (61%) with plasma cells constituting only 2% of nucleated cells in the marrow. Immunocytochemical studies at a reference laboratory showed positivity for CD138, CD56, and CD 20 with negativity for CD 38 suggestive of lymphoplasmacytic differentiation, while eyes findings including bilateral blindness and retinal features mimicking central retinal vein occlusion (CRVO) are suggestive of hyperviscosity retinopathy. Therefore, a diagnosis of lymphoplasmacytic lymphoma complicated by hyperviscosity retinopathy was made.

In the absence of an aphaeresis machine the patient was given four cycles of manual EBT to address the hyperviscosity as well as tablet allopurinol 100 mg/day, tablet chlorambucil 8 mg daily (0.1 mg/kg/day), tablet prednisolone 20 mg bd, and liberal fluids orally. Additionally, eye medications including gutt Zaditen Ophtha (Ketotifen Fumarate) every 8 hours in both eyes and tablet Diamox (acetazolamide) 250 mg every 12 hours were administered.

Following treatment, the patient's general condition improved with less body weakness and improved vision. The gum bleeding and pain also subsided. The VA initially improved to 6/18 (both eyes) about a month into the treatment and stabilized at 6/12 (RE), 6/36 (LE) five months into treatment without any improvement with pin hole test. Nevertheless, the near vision improved from N36 to N8 (RE) but remained N36 (LE) with +1.50 DS presbyopic correction. There was no improvement in distance vision following refraction. The IOP stabilized between 12 and 10 mmHg.

## 3. Discussion

Lymphoplasmacytic lymphomas (LPL) are rare and may present with uncommon and devastating symptoms. The weakness, headache, dizziness, bleeding, and visual disturbance which have been described in these patients are due to hyperviscosity caused by the IgM paraprotein produced by the abnormal cells resulting in changes in blood viscosity, abnormal rheology, and interference with coagulation factors and platelet function [3–5]. Waldenstrom's macroglobulinemia found in a subset of patients with lymphoplasmacytic lymphoma (LPL) is defined as LPL with bone marrow involvement and an IgM monoclonal gammopathy of any concentration [6]. The monoclonal gammopathy in our patient is IgG type, which is less common.

Visual disturbances affecting one or both eyes in patients with hyperviscosity due to IgM paraproteinemia have been reported [2, 5], but our patient is unique in that the paraprotein is IgG. Another risk factor for retinopathy/CRVO is uncontrolled mild hypertension due to hyperviscosity [9]. The patient had progressive bilateral visual loss although the onset was earlier in the left eye, but the difference could be anatomical since hyperviscosity retinopathy being of systemic origin is usually bilateral. CRVO associated with hypoproteinaemia (Waldenstrom macroglobulinemia or multiple myeloma) [1] may be reversible with plasmapheresis [10]. Improved vision was observed in this patient following manual EBT. Nevertheless; the visual recovery is better in the RE unlike the LE that had earlier onset of visual loss. The longer exposure of the LE to hypoxic condition due to severe anaemia on presentation (PCV 15%) might have caused more damage to the macular of the LE. Probably, fluorescein angiography (FA) would have illustrated some vascular filling differences between the eyes but this was not available in our clinic and the patient could not afford to have this investigation elsewhere. However, OCT demonstrated retinal haemorrhage, oedema, and diffuse photoreceptors damage which can explain poor distant (VA 6/36), near vision (N 36), and central visual field (CVF) defects worse on the LE. As there was no record of the patient's premorbid visual function test results, we relied on his subjective accounts. Remarkably, the patient's intraocular pressure (IOP) remains normal eight months after the onset of the visual loss and there was no evidence of rubeosis iridis. This may be due to the amelioration of hypoxic condition by interval manual EBT. Neovascularisation and elevated IOP are known complications in CRVO [11]. The patient was initially empirically placed on Diamox and Zaditen Ophtha; however, both were later discontinued as IOP remained normal and retinal findings including oedema and haemorrhages resolved.

Plasmapheresis, plasma exchange transfusion, and to a less extent chemotherapy have been reported in the treatment of hyperviscosity in Waldenstrom's macroglobulinemia/LPL with some improvement in clinical status and vision but not in all cases [4, 6, 8]. In the absence of an aphaeresis machine our patient (who has dependants including two wives and five children and is on a monthly salary of N71,000) was offered four cycles of manual exchange blood transfusion at total cost of N184, 460 followed by chemotherapy, that is, chlorambucil due to cost considerations (one USD exchanged for about 160 Naira).

## 4. Conclusion

Hyperviscosity symptoms in B-cell neoplasms are often associated with IgM paraprotein and are usually treated by aphaeresis. Our patient had IgG and could not receive that standard of care but improved on manual EBT and chemotherapy. The state of health infrastructure and cost of oncological care in sub-Saharan Africa has been adequately highlighted recently [12]. Our patient illustrates the challenge of providing basic diagnostic and support services for cancer patients in poor resource settings which requires concerted effort to overcome.

## Figures and Tables

**Table 1:** The results of initial blood investigations.

Indices	Patient value	Reference value
Blood indices		
WBC	4.07 × 10^9^	4–11 × 10^9^/L
PCV	15%	35–52%
MCV	78 fL	78–94 fL
MCH	25 pg	25–33 pg
MCHC	33 g/dL	31–37 g/dL
RDW	19.3%	11.6–14.0%
Platelets	198 × 10^9^/L	100–450 × 10^9^/L
ESR	>145 mm/hr	0–20 mm/hr

Serum electrolytes, urea and creatinine		
Na+	131 mmol/L	135–150 mmol/L
Bicarbonate	22 mmol/L	20–30 mmol/L
Creatinine	150 *μ*mol/L	50–106 *μ*mol/L

Liver function test		
Alkaline phosphatase	3 iu/L	≤129 iu/L
Random blood sugar	8.1 mmol/L	Fasting Blood Sugar −3.9–7.0 mmol/L
Serum cholesterol	1.6 mmol/L	<5.0 mmol/L
INR (PT)	1.42	0.9–1.3

## Abbreviations

BP: blood pressure
CRVO: central retinal vein occlusion
DS: Dioptre sphere
EBT: exchange blood transfusion
IOP: intraocular pressure
mmHg: millimetre of mercury
PTTK: Patial Thromboplastin Time Test with Kaolin
RE: right eye
LE: left eye
UATH: University of Abuja Teaching Hospital
VA: visual acuity.
